# High Concentrations of Methyl Fluoride Affect the Bacterial Community in a Thermophilic Methanogenic Sludge

**DOI:** 10.1371/journal.pone.0092604

**Published:** 2014-03-21

**Authors:** Liping Hao, Fan Lü, Qing Wu, Liming Shao, Pinjing He

**Affiliations:** 1 State Key Laboratory of Pollution Control and Resource Reuse, Tongji University, Shanghai, China; 2 Institute of Waste Treatment and Reclamation, Tongji University, Shanghai, China; 3 Centre for the Technology Research and Training on Household Waste in Small Towns & Rural Area, Ministry of Housing and Urban-Rural Development of P.R. China (MOHURD), Shanghai, China; Aix Marseille Université, France

## Abstract

To precisely control the application of methyl fluoride (CH_3_F) for analysis of methanogenic pathways, the influence of 0–10% CH_3_F on bacterial and archaeal communities in a thermophilic methanogenic sludge was investigated. The results suggested that CH_3_F acts specifically on acetoclastic methanogenesis. The inhibitory effect stabilized at an initial concentration of 3–5%, with around 90% of the total methanogenic activity being suppressed, and a characteristic of hydrogenotrophic pathway in isotope fractionation was demonstrated under this condition. However, extended exposure (12 days) to high concentrations of CH_3_F (>3%) altered the bacterial community structure significantly, resulting in increased diversity and decreased evenness, which can be related to acetate oxidation and CH_3_F degradation. Bacterial clone library analysis showed that syntrophic acetate oxidizing bacteria *Thermacetogenium phaeum* were highly enriched under the suppression of 10% CH_3_F. However, the methanogenic community did not change obviously. Thus, excessive usage of CH_3_F over the long term can change the composition of the bacterial community. Therefore, data from studies involving the use of CH_3_F as an acetoclast inhibitor should be interpreted with care. Conversely, CH_3_F has been suggested as a factor to stimulate the enrichment of syntrophic acetate oxidizing bacteria.

## Introduction

Methane is both a greenhouse gas and a bioenergy gas. The metabolism of methane formation has recently generated a great deal of interest due to the greenhouse effect and the energy crisis. Methane formation can be realized via acetoclastic or hydrogenotrophic methanogenesis, which predominates in anaerobic digestion of biomass. The ratio of the two pathways can be influenced by various environmental factors such as pH, acetate or ammonia concentrations [1]–[2], the oxidation state of labile organic carbon and competing microbial processes [3].

To better understand the controlling factors for CH_4_ production in anoxic or anaerobic environments, methyl fluoride (CH_3_F) has been frequently used and regarded as a specific inhibitor for acetoclastic methanogenesis [3]–[7]. The inhibitory effect of CH_3_F has been validated in pure culture studies [8], as well as in rice fields [4], [6], [9], [10], lake sediments [11] and anaerobic reactors [12], [13]. Inhibitor experiments combined with isotope probing and signature were conducted to confirm the specific inhibition on the acetoclastic pathway [4], [10]–[13]. According to the results of the aforementioned experiments, a CH_3_F concentration of 1–3% was suggested to be used in various environments including the rhizosphere [4], [10], lake sediments [11] and anaerobic digestors under thermophilic [12] and mesophilic [13] conditions to specifically suppress the acetoclastic pathway without inhibiting the hydrogenotrophic pathway.

To further investigate the effect of CH_3_F on the microbial community, the inhibition of CH_3_F (approx. 1%) on the growth of acetotrophic *Methanosaeta* sp. and *Methanosarcina* sp. was also confirmed in pure cultures [8] and in the rhizosphere [6]. Other microbes, including homoacetogenic, sulfate reducing bacteria (e.g., *Desulfotomaculum* spp.) and fermentative bacteria (e.g., *Acetobacterium* spp.), as well as a methanogenic mixed culture based on hydrogen syntrophy, were not inhibited [8]. Thus, it is often assumed that other organisms in anoxic or anaerobic mixed cultures are not affected by CH_3_F, and data are interpreted accordingly to infer the roles of methanogens and other microorganisms in the consortium.

However, microbial populations may act differently in complicated environmental microcosms compared with that in pure culture due to redistribution of the substrates and competition among various populations. Daebeler et al. [3] recently demonstrated that CH_3_F affects methanogenic activity rather than community composition of methanogenic archaea in a rice field soil, which was in accordance with the findings by Hao et al. [14]. Conversely, the syntrophic acetate oxidizing (SAO) bacteria and hydrogenotrophic methanogens were in higher abundance in the CH_3_F (3%) treatments after an attack by pH disturbance [1]. Moreover, CH_3_F can inhibit CH_4_- or ammonium-oxidizing bacteria [15], [16]. Therefore, the assumption that bacteria are not affected by CH_3_F is questionable, and further research is necessary to better understand the influence of CH_3_F on the microbial community structure in mixed cultures, especially on bacterial community structure.

Additionally, the above conclusions are only based on experiments conducted at a relatively low CH_3_F concentration of 0–3%, while nearly no information regarding changes in microbial community structure has been reported for wider ranges of CH_3_F concentration. Considering that the appropriate dose of CH_3_F differs under different environmental conditions [13], the influence of higher concentrations of CH_3_F on micro-ecology needs to be studied.

In the present study, to appropriately control the application of CH_3_F for analysis of methanogenic pathways, the influence of 0–10% CH_3_F on the bacterial and archaeal community structure was investigated using denaturing gradient gel electrophoresis (DGGE). Since bacterial DGGE fingerprints were found to change significantly at high CH_3_F levels, while archaeal ones were not, a bacterial clone library was constructed at CH_3_F (10%) to probe into the mechanism for this alteration. Process measurement and isotope signature were applied to identify the inhibitory effect in the inhibitor experiment. Additional evidence was presented that, extended exposure to high concentration of CH_3_F affected bacterial community structure in a thermophilic methanogenic sludge.

## Materials and Methods

### Experimental Set-up

Freshly collected methanogenic granular sludge, which was previously cultivated at 55°C in an anaerobic sequenced batch reactor, was used as the inoculum. The inoculum was added to serum bottles (250 mL) with 100 mL basal medium at a volatile solid (VS) concentration of about 3 g L^−1^. The basal medium contained per L: 1.0 g NH_4_Cl, 0.4 g K_2_HPO_4_·3H_2_O, 0.2 g MgCl_2_·6H_2_O, 0.08 g CaCl_2_·2H_2_O, 0.1 g yeast extracts, 0.2 g Na_2_S·9H_2_O, 10 mL trace element solution (containing per L: 400 mg FeCl_2_·4H_2_O, 19 mg H_3_BO_3_, 100 mg ZnCl_2_, 20 mg CuCl_2_·2H_2_O, 100 mg MnCl_2_·4H_2_O, 10 mg Na_2_MoO_4_·4H_2_O, 90 mg AlCl_3_·6H_2_O, 170 mg CoCl_2_.6H_2_O, 20 mg NiCl_2_·6H_2_O, 194 mg Na_2_SeO_3_·5H_2_O, 1.0 g EDTA-2Na) and 5 mL stock vitamin solution (containing per L: 10 mg biotin, 50 mg pyridoxinHCl, 25 mg thiamineHCl, 25 mg D-calcium pantothenate, 10 mg floic acid, 25 mg riboflavin, 25 mg nicotinic acid, 25 mg 4-Aminobenzic acid and 0.5 mg vitamin B_12_). Sodium acetate was then added as the substrate to an initial concentration of 54 mM. Bicarbonate buffer was used and the pH of the bulk liquid was adjusted to 6.8 with 1 M HCl. Reagents were provided by Sinopharm Chemical Reagent Co., Ltd. (Shanghai, China).

After the headspace was filled with a N_2_/CO_2_ (4/1, V/V) gas mixture (1 atm), CH_3_F (99%, Shanghai Chunyu Special Gas Co., Ltd, China) was injected into the headspace and then mixed by manual shaking. A total of 12 different reactors were set up at different initial CH_3_F concentrations of 0, 0.5%, 1.0%, 1.5%, 2.0%, 2.5%, 3.0%, 4.0%, 5.0%, 6.0%, 8.0% and 10% (V/V). The sludge was incubated statically at 55°C in the dark for 12 days, during which time period liquid and gas samples were taken periodically for analysis.

### Physical-chemical Analysis of the Samples

Gas composition, including CH_4_, CO_2_, H_2_ and CH_3_F, was periodically monitored. The stable carbon isotopic compositions of CH_4_ (*δ* ^13^CH_4_) and CO_2_ (*δ* ^13^CO_2_) in the headspace were measured on day 6. The liquid samples were analyzed for pH, total organic carbon, total inorganic carbon and volatile fatty acids. Samples were analyzed using methods that have been described in detail elsewhere [13].

### DNA Extraction and PCR-DGGE for Bacteria and Archaea

Sludge samples were collected at the end of the incubation period on day 12 and DNA was extracted from the seed and incubated sludge. Polymerase chain reaction (PCR), and DGGE of the PCR products for bacteria and archaea were conducted as previously described [12]. Sequencing of single DGGE bands and the subsequent analyses were conducted using the methods described by Ye et al. [17].

### Establishment of Bacterial 16S rRNA Gene Library

A bacterial clone library was generated from PCR-amplified 16S rRNA gene using bacterial primers 27 f (5′-AGA GTT TGA TCC TGG CTC AG-3′) [18] and 1492r (5′-GGT TAC CTT GTT ACG ACT T-3′) [18]. Each PCR reaction (50 μL) contained 1 μL of template, 10 pmol of each primer, 5 μL of 10 × Red Hot PCR buffer, 100 μmol of dNTPs, 20 mmol of MgCl_2_ and 1 U of Taq DNA polymerase (Shanghai Biocolor BioScience and Technolgy Company, China). PCR amplification was conducted as follows: 94°C for 3 min followed by 30 cycles of denaturation at 94°C for 45 s, annealing at 55°C for 45 s, and extension at 72°C for 1.5 min and then a single final extension at 72°C for 10 min. PCR reactions were performed on a Mastercycler®ep realplex^2^ qPCR thermal cycler (Eppendorf AG, Hamburg, Germany).

To minimize PCR artifacts, reconditioning PCR was conducted according to the method described by Thompson et al. [19] after initial amplification of the bacterial 16S rRNA gene. Briefly, the PCR-amplified reaction was diluted 10-fold in a fresh reaction mixture of the same composition and cycled three times using this program. ssDNA and heteroduplex DNA were minimized by adding excess primer during the reaction [20].

### Cloning and Sequencing

Bacterial reconditioning PCR products of 0.2 pmol were electrophoresed on 1.0% agarose and bands of the correct size (>1.5 kb for bacteria) were purified using an EZ-Spin Column DNA Gel Extraction Kit (Sangon Biotech Co., China).

Finally, the purified product was cloned into the pUCm−T vector (Sangon Biotech Co., China) following the manufacturer's instructions. The ligated products were transformed into *Escherichia coli* competent cells (SK2301, Sangon Biotech Co., China) with ampicillin and blue/white screening, after which positive clones were arrayed in 96-well plates and stored at −80°C for long-term storage. Plasmid inserts were checked by PCR amplification using an M13 PCR set and 103 bacterial positive insert-containing clones were randomly selected for gene sequencing. The template DNA was prepared from overnight cultures of selected clones using a UNIQ-10 Column Plasmid Mini-Preps Kit (Sangon Biotech Co., China), sequencing was performed on an ABI 3730 DNA sequencer (Applied Biosystems, CA, USA) with BigDye Terminator chemistry according to the manufacturer's instruction.

### Sequence Analysis

Sequences were analyzed manually to remove vector and ambiguous sequences at the ends by scanning the individual chromatograms using Chromas software ver.2.23 (Technelysium, Shanghai, China). Chimeras were checked by the CHIMERA_CHECK program [21] in Ribosomal Database Project (RDP) firstly, and were further firmed by DECIPHER and Bellerophon program on the Greengenes website [22]. After eliminating low-quality sequences and chimeric sequences, 89 sequences were used for the subsequent analyses. All reference sequences were obtained from the GenBank (NCBI) and RDP. Bacterial nucleotide sequences obtained in this study are available in the GenBank database under accession numbers: KF990054−KF990120 and KJ003859−KJ003880.

### Data Processing

The inhibition efficiency and apparent stable carbon isotope fractionation factor (*α*_c_) were calculated according to the methods described by Hao et al. [13]. Diversity and evenness of the microbial community structure based on the DGGE profiles were assessed using the methods as described previously [14], [23]–[26].

## Results and Discussion

### Metabolites and Inhibitory Effect

**Figure 1:**
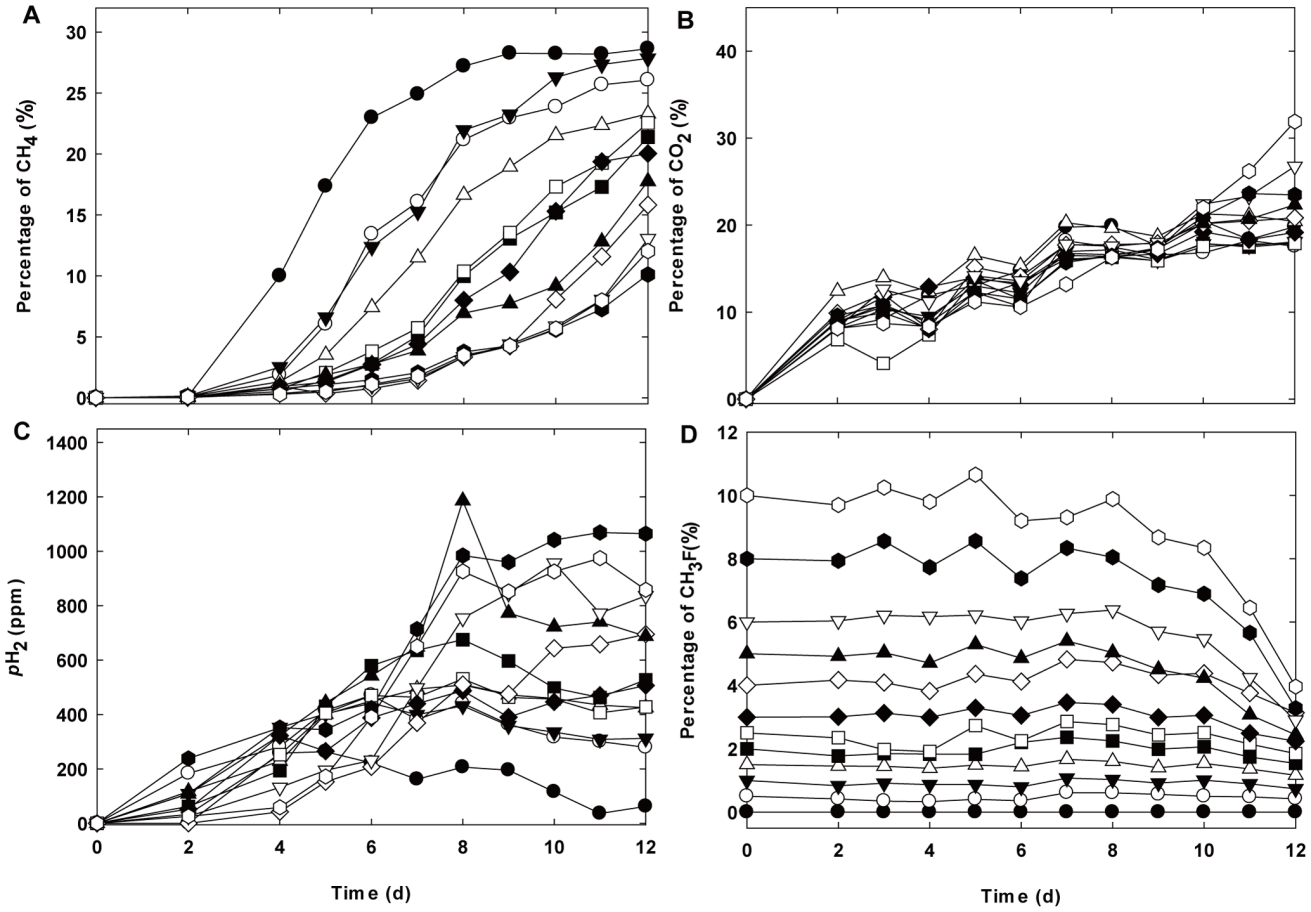
Temporal changes in gas composition in the headspace. (A) percentage of CH_4_, (B) percentage of CO_2_, (C) H_2_ partial pressure and (D) percentage of CH_3_F in different reactors. 0% CH_3_F (solid circle), 0.5% CH_3_F (open circle), 1.0% CH_3_F (solid inverted triangle), 1.5% CH_3_F (open triangle), 2.0% CH_3_F (solid square), 2.5% CH_3_F (open square), 3.0% CH_3_F (solid diamond), 4.0% CH_3_F (open diamond), 5.0% CH_3_F (solid triangle), 6.0% CH_3_F (open inverted triangle), 8.0% CH_3_F (solid hexagon), 10.0% CH_3_F (open hexagon).

**Figure 2:**
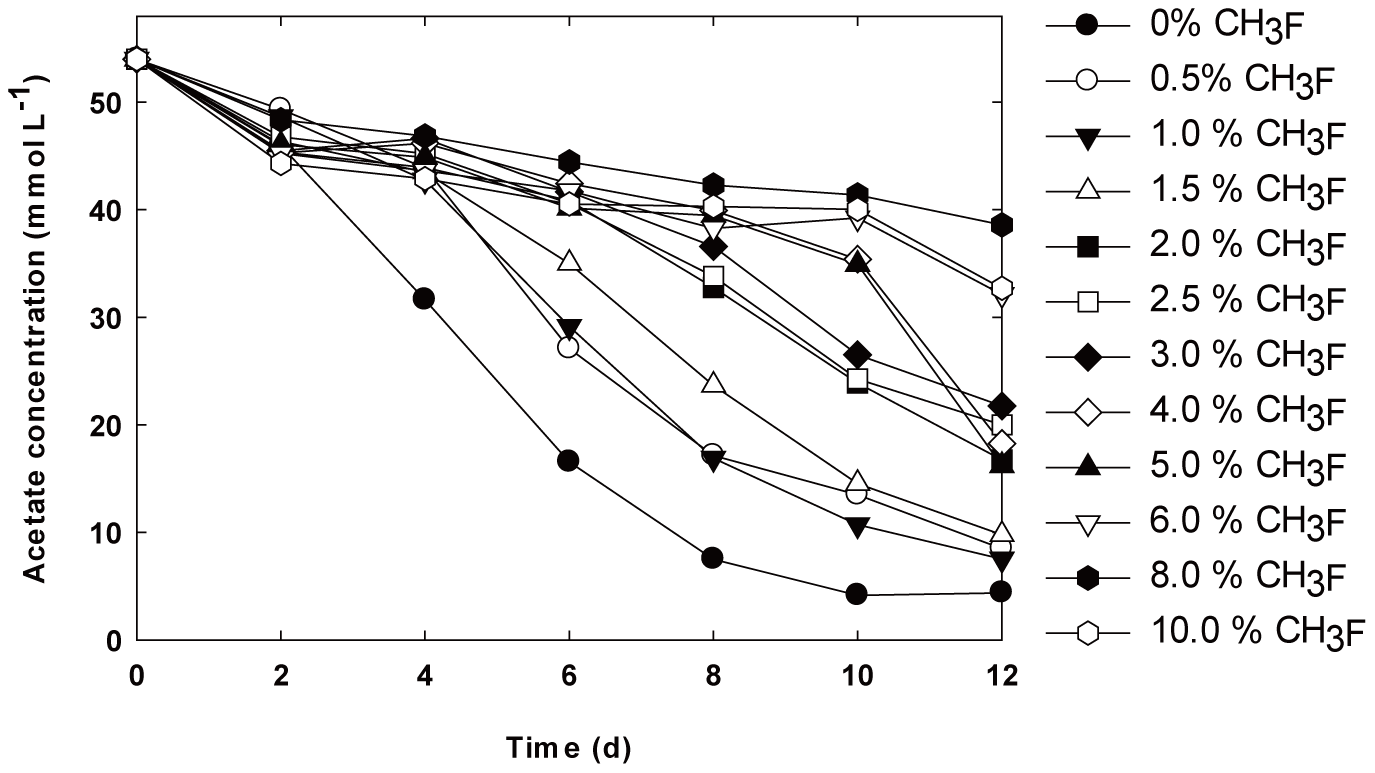
Temporal changes in acetate concentration under different CH_3_F levels. 0% CH_3_F (solid circle), 0.5% CH_3_F (open circle), 1.0% CH_3_F (solid inverted triangle), 1.5% CH_3_F (open triangle), 2.0% CH_3_F (solid square), 2.5% CH_3_F (open square), 3.0% CH_3_F (solid diamond), 4.0% CH_3_F (open diamond), 5.0% CH_3_F (solid triangle), 6.0% CH_3_F (open inverted triangle), 8.0% CH_3_F (solid hexagon), 10.0% CH_3_F (open hexagon).

As shown in the profiles of the biogas composition (**Figure 1**) and acetate concentration (**Figure 2**), the inhibitory effect of CH_3_F could be clearly seen in the kinetics of CH_4_ formation and acetate degradation when compared with that of the control experiment in which no CH_3_F was added. Increasing CH_3_F concentrations led to greater inhibition of mineralization of acetate and higher hydrogen partial pressure in the headspace.

However, along with incubation time, the inhibitory effect was not consistent. CH_4_ production was obviously accelerated after 7 days of incubation at high CH_3_F concentrations (≥ 3%) when compared with that observed in the earlier period. Since an apparent decrease in CH_3_F concentration was observed after 7 or 8 days (initial CH_3_F concentrations ≥ 3%), the resumption of methanogenic activity could be related to the loss of CH_3_F. To evaluate the acute toxicity of CH_3_F, the degree of inhibition was quantified at day 6, with a consistent inhibitory effect occurring during the period of 0–6 days. The fitted dose-response curve had ED_90_ (effective dose for 90% inhibition on methanogenic activity) concentrations of 3.8% CH_3_F. When the CH_3_F concentration was as high as 6–10%, the anaerobic consortium still showed 5% methanogenic activity of that displayed in the control experiment (**Figure S1**).

Higher resistance of hydrogenotrophic methanogens to CH_3_F has frequently been reported [4], [6], [8], [13]. In a previous experiment [3], hydrogenotrophic methanogenesis was found to be unaffected, even at 4% CH_3_F. Thus, the hydrogenotrophic pathway was suggested to contribute to partial CH_4_ formation in the current study.

### Isotopic Signature

**Figure 3:**
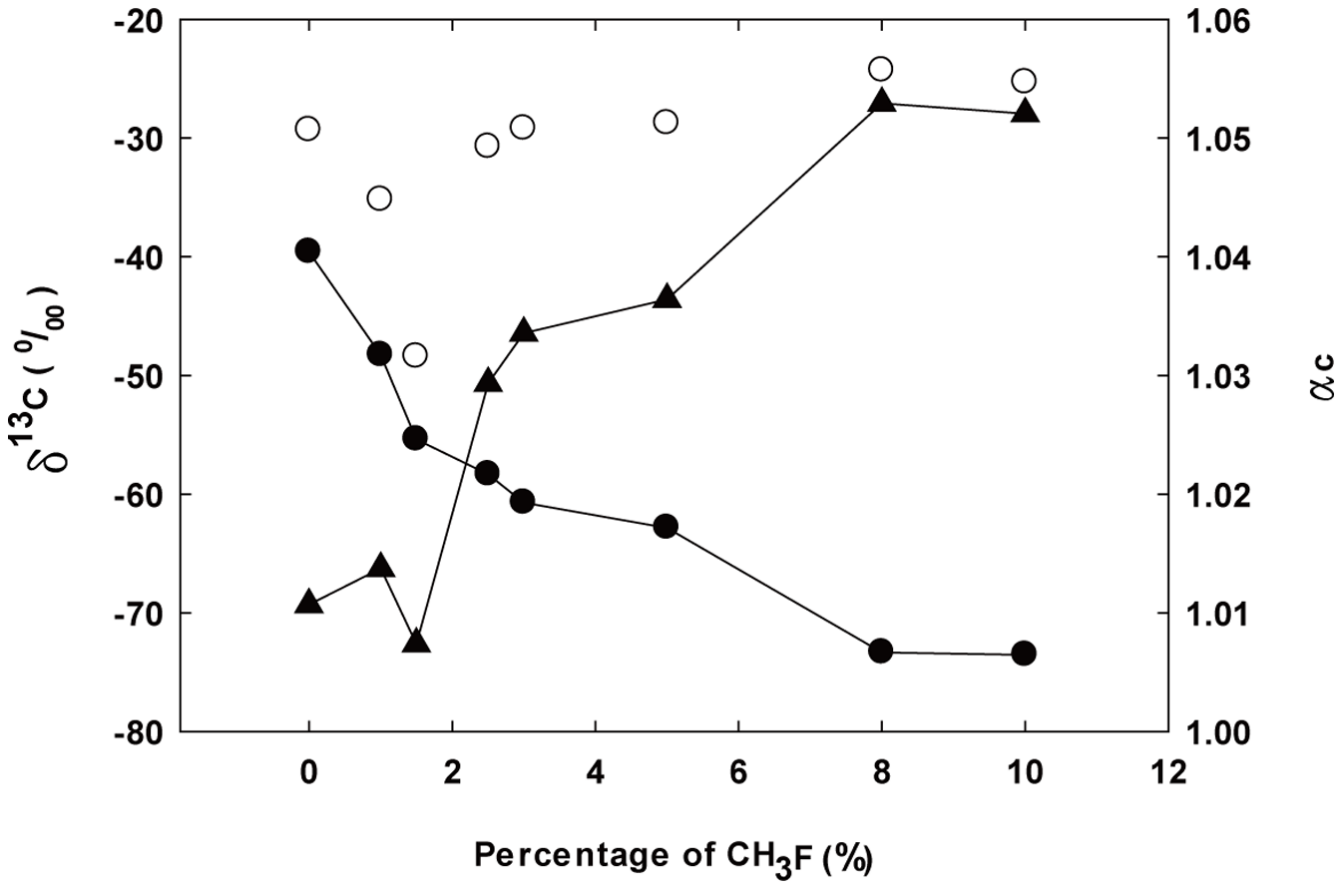
Temporal changes in *δ* ^13^CH_4_, *δ* ^13^CO_2_ and *α*_c_ at day 6 under different CH_3_F levels. *δ* ^13^CH_4_ (solid circle), *δ* ^13^CO_2_ (open circle), *α*_c_ (solid triangle).

The influence of CH_3_F on methanogenic pathways can also be elucidated by the change in isotopic fractionation factor *α*_c_ along with the elevated CH_3_F concentration (**Figure 3**). *α*_c_ was around 1.01 at CH_3_F concentrations of 0–1.5%, indicating a predominant pathway of acetoclastic methanogenesis. *δ* ^13^CH_4_ gradually decreased to −62.8‰ and *δ* ^13^CO_2_ increased to −28.7‰, resulting in an increase of *α*_c_ to about 1.04 at CH_3_F concentrations of 2.5–5%. An increase of the contribution of the hydrogenotrophic pathway was thus indicated, and even dominance could be possible as reported by Hao et al. [13]. As the CH_3_F concentration was further increased to 8–10%, *δ* ^13^CH_4_ and *δ* ^13^CO_2_ quickly decreased to −73.4‰ and increased to −24.3‰, respectively, with *α*_c_ increasing to 1.05. Hence, the hydrogenotrophic pathway might be further concentrated, or the functional microbial players could have changed under higher stress from 8–10% CH_3_F, resulting in different isotopic fractionation [5].

### Bacterial and Archaeal DGGE Fingerprinting

Use of CH_3_F (1 kPa, around 1% in the headspace) is generally assumed to eliminate archaea while having little effect on bacteria [6]–[8]. However, DGGE analyses in this study revealed additional information.

**Figure 4:**
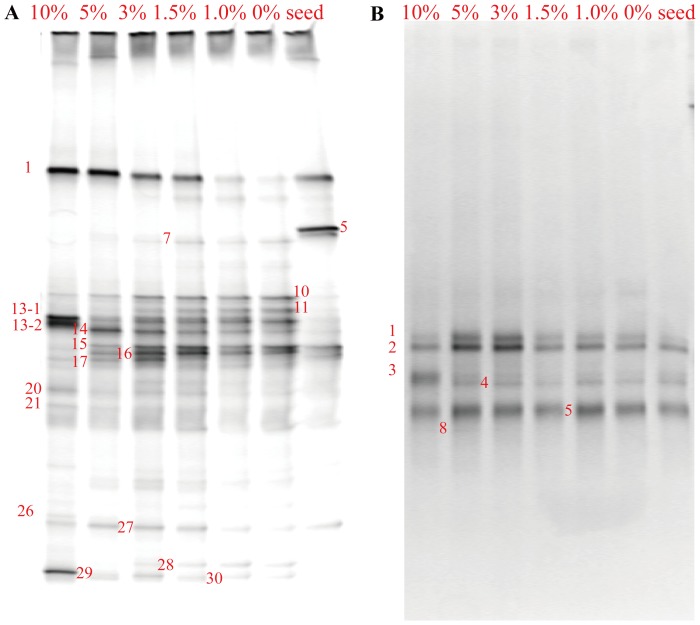
Denaturing gradient gel electrophoresis (DGGE) fingerprints of the microbial communities. (A) bacterial and (B) archaeal communities in the sludge after incubation under different CH_3_F levels.

As shown in **Figure 4A**, long-term (12 days) exposure to various levels of CH_3_F (0–10%) led to surprising alterations in the bacterial community. The bacterial DGGE profiles demonstrated a gradual change at high CH_3_F concentrations (1.5–10%), while they stayed stable at lower CH_3_F levels (0–1.5%). It can be seen that, along with the elevated CH_3_F concentration, bands 14, 15 16 and 27 were first promoted (CH_3_F 0–3%) and then eliminated (CH_3_F 3–10%). Band 17 initially appeared at a CH_3_F of 3%, and then gradually faded out at higher CH_3_F concentrations. New bands 13–2, 26 and 29 appeared and bands 13–1, 13–2 and 29 had quite high intensity at 10% CH_3_F. Correspondingly, in this treatment, the diversity and evenness of the bacterial community structure increased to 2.88 and decreased to 0.86, respectively, when compared with the treatments using lower CH_3_F levels (**Table 1**). These findings indicate that more bacterial populations exist under these conditions, but that only a few are active functional players related to the metabolism of acetate or CH_3_F.

**Table 1:** Summarized information of denaturing gradient gel electrophoresis (DGGE) profiles in the present study.

Sample	Bacteria				Archaea			
	*H* a	*E* b	*S* c	20% PL Evenness^d^	*H*	*E*	*S*	20% PL Evenness
Seed	2.41	0.89	15	0.49	1.56	0.97	5	0.27
0% CH_3_F	2.56	0.85	20	0.55	1.90	0.98	7	0.29
1% CH_3_F	2.69	0.91	19	0.44	1.73	0.97	6	0.30
1.5% CH_3_F	2.87	0.92	23	0.46	1.37	0.99	4	0.24
3% CH_3_F	2.86	0.90	24	0.47	1.73	0.97	6	0.30
5% CH_3_F	2.76	0.89	22	0.47	1.71	0.95	6	0.31
10% CH_3_F	2.88	0.86	28	0.57	1.90	0.97	7	0.28

a Shannon index of general diversity (*H*) as described by Tiwar et al. [23].

b The indice (*E*) to evaluate the evenness of microbial structure as calculated by Pielou [24].

c Based on the Pareto principle, the cumulative y axis value (in this case the proportion of intensities) corresponding with the 20% level on the x axis (in this case the cumulative proportion of band numbers) (20%PL Evenness) used to evaluate the Lorenz curves by Wittebolle et al. [25].

d Richness (*S*) of the bacterial and archaeal communities as described by Chau et al. [26], which is the number unique taxa present in the sample (here represented by the number of bands in the DGGE lanes).

Sequencing of these bands showed that bands 13–1 and 29 were affiliated with the *Firmicutes* phylum, while band 14 corresponded to the *Bacteroidetes* phylum (**Table 2**). Most of the other bands (such as band 10 and 16), however, can only match to the uncultured bacteria, except that band 7 was assigned to the *Thermotogae* phylum and band 17 belonged to the *Synergistetes* phylum. According to previous studies, 4 of the 5 identified SAO bacteria belong to the *Firmicutes* phylum [27], including the *Clostridium ultunense* strain BST [28], *Syntrophaceticus schinkii* [29], *Tepidanaerobacter acetatoxydans* [30] and *Thermacetogenium phaeum* strain PB [31], while *Thermotoga lettingae* strain TMO [32] is affiliated with the *Thermotogae* phylum. Uncultured bacteria in the *Thermoanaerobacteriaceae* family were also found to possess the SAO function [33]. Thus, the members of *Firmicutes* and *Thermotogae* in the microcosm could be related to the SAO bacteria. Sequencing of some other bands (like bands 15 and 27) failed; hence, no specific information was available for these bands.

**Table 2:** Phylogenetic sequence affiliation of amplified 16S rRNA gene sequences excised from bacterial denaturing gradient gel electrophoresis (DGGE) gels.

Band ID	Length (bp)	Phylogenetically most closely related organism (Accession No.)	Max identity	Phylum
1	190	Uncultured *Bacteroidetes* bacterium (CU918707.1)	100%	*Bacteroidetes*
5	204	Uncultured bacterium (JN707963.1)	99%	unclassified
7	204	*Thermotogaceae* bacterium (JX088344.1)	99%	*Thermotogae*
10	179	Uncultured bacterium (GQ423781.1)	100%	unclassified
12	200	Uncultured bacterium (EF515355.1)	98%	unclassified
13–1	176	*Thermoanaerobacteriaceae* bacterium (GU129121.1)	100%	*Firmicutes*
13–2	199	Uncultured bacterium clone thermophilic_alkaline-14 (GU455254.1)	97%	unclassified
14	201	*Bacteroidetes* bacterium (JN836378.1)	100%	*Bacteroidetes*
16	182	Uncultured bacterium (JQ519265.1)	100%	unclassified
17	216	*Synergistetes* bacterium (JX473564.1)	100%	*Synergistetes*
20	198	*Bacteroidetes* bacterium (JN836378.1)	100%	*Bacteroidetes*
21	195	Uncultured bacterium (JQ316632.1)	98%	unclassified
26	212	Uncultured bacterium (HQ267061.1)	97%	unclassified
28	176	Uncultured bacterium (HE804957.1)	100%	unclassified
29	177	Uncultured *Firmicutes* bacterium (JQ433814.1)	99%	*Firmicutes*
30	193	Uncultured bacterium (AB374122.1)	98%	unclassified

Incubation at CH_3_F of 0–10% did not alter the archaeal DGGE profiles significantly, which is in accordance with the results of a study by Daebeler et al. [3], who found that the suppression of acetoclastic methanogenesis by CH_3_F caused little differences in the community composition of active methanogenic archaea in a rice field soil. While, it could be seen that, the intensity of bands 1 and 2 slightly increased at CH_3_F of 3–5%. Sequencing results showed that bands 1 (98%) and 2 (99%) corresponded to uncultured archaea in the *Methanobacterium* genus (**Table 3**), while band 5 (99%) was closely related to the acetotrophic *Methanosaeta* genus. These findings suggested that hydrogenotrophic methanogens were enriched to some extent in response to high concentrations of CH_3_F.

**Table 3:** Phylogenetic sequence affiliation of amplified 16S rRNA gene sequences excised from archaeal denaturing gradient gel electrophoresis (DGGE) gels.

Band ID	Length (bp)	Phylogenetically most closely related organism (Accession No.)	Max identity	Order
1	151	Uncultured *Methanobacterium* sp. (AB602627.1)	98%	*Methanobacteriales*
2	152	Uncultured *Methanobacterium* sp. (JX576095.1)	99%	*Methanobacteriales*
4	153	*Methanobacterium kanagiense* (AB368917.1)	97%	*Methanobacteriales*
5	152	*Methanosaeta* sp. (JX088310.1)	99%	*Methanosarcinales*
8	152	*Methanothermobacter thermautotrophicus* (NR_074260.1)	95%	*Methanobacteriales*

### Bacterial Community Confirmed by Clone Library

Acetate metabolism can proceed via SAO in methanogenic systems without electron accepters such as sulphate, nitrate and chlorate. Analysis of the DGGE profiles indicated that the highly concentrated bands might be related to the SAO bacteria at high CH_3_F concentrations. To obtain in-depth knowledge regarding the bacterial community under these conditions, a clone library was constructed for the microcosm adapted to the treatment with 10% CH_3_F, and the results confirmed the enrichment of SAO bacteria under pressure from high concentrations of CH_3_F.

The 89 sequences of 16S rRNA genes were classified into 29 operational taxonomic units (OTUs) as shown in **Table S1**. Overall, 87% of the sequences were classified as *Firmicutes*, 4% as *Bacteroides* and 4% as *Synergistetes*. The taxonomic information of 13 representative OTUs, which were phylogenetically different, was listed in **Table 4**.

**Table 4:** Taxonomic relationship of bacterial 16S rRNA gene sequences in 13 representative OTUs from 10% CH_3_F treatment compared (BLAST) with public databases (RDP and NCBI).

OTU	% of Total	Length (bp)	Phylogenetically most closely related organism (Accession No.)	Accession No.	Phylum	Similarity (%)	Function	Source
BS01	46	1593	*Thermacetogenium phaeum* (NR_074723.1)	KF990054	*Firmicutes*	94%	Syntrophic acetate oxidation	DSM 12270
BS24	2	1504	*Syntrophaceticus schinkii* (EU386162.1)	KF990067	*Firmicutes*	94%	Syntrophic acetate oxidation	Mesophilic anaerobic filter
BS21	0	1502	Uncultured *Thermacetogenium* sp. (HQ183800.1)	KF990065	*Firmicutes*	94%	Syntrophic acetate oxidation	Leachate sediment in landfill
BS28	4	1485	*Lutispora thermophila* (NR_041236.1)	KF990071	*Firmicutes*	97%	Protein-fermenting	Strain EBR46
BS84	1	1486	*Clostridium aldrichii* (X71846.1)	KF990107	*Firmicutes*	93%	Cellulolytic	DSM 6159
BS66	1	1487	Uncultured bacterium (JF808030.1)	KF990092	*Firmicutes*	99%	—	Produced fluid from Yabase oilfield
BS22	4	1296	*Anaerobaculum mobile* (CP003198.1)	KJ003866	*Synergistetes*	97%	Peptide-fermenting	DSM 13181
BS40	1	1257	*Bacteroidetes* bacterium (AY548787.1)	KF990076	*Bacteroidetes*	98%	—	Fluidized-bed reactors: acidic wastewater
BS70	1	1503	Uncultured bacterium (AB669265.1)	KF990096	*Bacteroidetes*	99%	—	Anaerobic digester sludge
BS79	1	1488	Uncultured *Cytophagales* bacterium (FJ516908.1)	KF990102	*Bacteroidetes*	97%	—	The semiarid ‘Tablas de Daimiel National Park’ wetland
BS96	1	1488	Uncultured bacterium (AB192126.1)	KJ003880	*Chlorobi*	96%	—	Gut homogenate of termites
BS94	1	1494	Uncultured bacterium (FJ462092.1)	KJ003878	unclassified	99%	—	Anaerobic reactor: effluent from the chemical industry
BS87	1	1489	Uncultured bacterium (EF205585.1)	KF990110	unclassified	99%	—	Geothermal spring mat

OTU, operational taxonomic unit.

In the sequences assigned to the *Firmicutes* phylum, the most abundant group of OTUs, with BS01 as the most representative, matched the pure culture (DSM 12270) of *Thermacetogenium phaeum* (NR_074723.1) with a similarity of 93–94%, which occupied 61% of the total sequences. Another group of OTUs, with BS24 as the most representative, were closely related to *Syntrophaceticus schinkii* (EU386162.1), which was isolated from a mesophilic anaerobic filter [29]. *Thermacetogenium phaeum* was reported as a SAO bacterium grown under thermophilic conditions [31]. *Syntrophaceticus schinkii* also oxidize acetate to support their growth under mesophilic condition [29], but has ever been found in thermophilic reactors as well [2]. It was thus indicated that, bacteria with SAO function dominated the methanogenic sludge incubated with 10% CH_3_F. Notably, there were still 5 OTUs (with BS21 as the most representative) contributing to 9% of the total sequences, which were closely affiliated to the uncultured *Thermacetogenium* sp. (HQ183800.1) [34]. These bacteria populations could be new, previously undescribed species with SAO function grown under thermophilic conditions.

The other OTUs, including BS22, BS28 and BS84, were related to fermentative bacteria functioning at protein and cellulose degradation, which accounted for 4%, 4% and 1% of the total sequences, respectively. These OTUs might represent some of the aboriginal bacterial populations related with endogenous metabolism and cellulose degradation, since the sludge originated from an anaerobic digester treating wastewater from a paper mill before acclimatization. Most other OTUs could only be matched to uncultured bacteria without clearly defined functions.

### Influence of CH_3_F on Microbial Community

Analyses of the microbial community structure demonstrated that long-term exposure to high concentrations of CH_3_F (10%) induced the enrichment of SAO bacteria, which has seldom been reported in the literature. *Thermacetogenium phaeum*-related bacteria predominated the microcosm (**Table 4**), and diversity of the bacterial community structure increased while the evenness decreased (**Table 1**). Faster growth of SAO bacteria was also indicated in CH_3_F treatments (3%) after an attack by high pH in a previous study [1]. Based on these findings, it could be speculated that high concentrations of CH_3_F strongly inhibited the acetotrophic methanogens [8]; without their competition for acetate, SAO bacteria that were unaffected or less affected by CH_3_F became active and enriched. The consequently promoted SAO reaction generated more H_2_ and CO_2_, resulting in increased hydrogen partial pressure (**Figure 1C**). The hydrogenotrophs might be thus activated, leading to stronger isotope fractionation (**Figure 3**). Methanogenic activity resumed along with the incubation time and possibly with the decreasing CH_3_F concentrations (**Figure 1D**). By contrast, the microbial community structure did not change obviously at CH_3_F concentrations of 0–1.5%, which was similar to the results of an experiment conducted by Hao et al. [14]. These results indicated that CH_3_F usage should be carefully controlled to prevent significant changes in the microbial community structure if the methanogenic function of the microbial community is intended to be evaluated under *in-situ* conditions.

However, from the perspective of bioaugmentation, use of high concentrations of CH_3_F may be an efficient tool to stimulate the enrichment of SAO bacteria. Then, the acclimatized consortium can be used as inoculum to address the inhibition problems that occur in anaerobic digestion processes as a result of ammonia or other acetoclast inhibitors [35].

### Biodegradation of CH_3_F

The CH_3_F concentration decreased quickly in the thermophilic methanogenic system, especially at high initial CH_3_F levels of 4–10%, which decreased to 3–4.5% after 7 or 8 days of incubation (**Figure 1D**). Similar phenomena were observed in the previous experiments [4], [13], [36]. It is thus suggested that microbial degradation of CH_3_F occurs in anaerobic and anoxic systems. Although new DGGE bands appeared after 8 days of incubation under high levels of CH_3_F, the phylogenetic affiliations of the CH_3_F-degrading microbes that may be present are still not clear. Therefore, further research using stronger analytical tools such as stable isotope probing are warranted.

## Conclusions

The influence of 0–10% CH_3_F on the microbial community in a thermophilic methanogenic sludge was investigated. The results suggested that, CH_3_F acted specifically on acetoclastic methanogenesis, and the inhibitory effect stabilized at an initial concentration of 3–5%, with around 90% of the total methanogenic activity being suppressed and a characteristic of hydrogenotrophic pathway in isotope fractionation occurring. However, extended exposure (12 days) to high concentrations of CH_3_F (>3%) altered the bacterial community structure significantly, with the diversity increased while the evenness decreased. These changes were likely related to acetate oxidation and CH_3_F degradation. Bacterial clone library analysis showed that, syntrophic acetate oxidizing bacteria (*Thermacetogenium phaeum*) were highly enriched under the pressure from 10% CH_3_F, but the methanogenic community did not change obviously. Thus, excessive use of CH_3_F over a long term can change the composition of the bacterial community. Therefore, data from studies involving the use of CH_3_F as an acetoclast inhibitor should be interpreted with care. However, from the perspective of bioaugmentation, CH_3_F can be considered as a factor that stimulates the enrichment of SAO bacteria.

## Supporting Information

Figure S1 **Dose–response curve for the inhibitory effect of CH_3_F.** The solid line describes the inhibition of methanogenesis as a function of the initial CH_3_F concentration for anaerobic granules.
(DOCX)

Table S1 **Bacterial community analyzed by clone library.** Taxonomic relationship of bacterial 16S rRNA gene sequences in 29 OTUs from 10% CH_3_F treatment compared (BLAST) with public databases (RDP and NCBI).
(DOC)
