# Supplementary material for: High Concentrations of Methyl Fluoride Affect the Bacterial Community in a Thermophilic Methanogenic Sludge
Source: PLoS One. 2014 Mar 21;9(3):e92604. doi: 10.1371/journal.pone.0092604 (PMC3962445; doi:10.1371/journal.pone.0092604)
Supplement: Table S1 — Bacterial community analyzed by clone library. Taxonomic relationship of bacterial 16S rRNA gene sequences in 29 OTUs from 10% CH3F treatment compared (BLAST) with public databases (RDP and NCBI). (DOC) [file pone.0092604.s002.doc]

**Table S1. Bacterial community analyzed by clone library.** Taxonomic relationship of bacterial 16S rRNA gene sequences in 29 OTUs from 10% CH_3_F treatment compared (BLAST) with public databases (RDP and NCBI)

| OTU | % of Total | Length (bp) | Phylogenetically most closely related organism (Accession No.) | Accession No. | Phylum | Similarity (%) | Function | Source |
| --- | --- | --- | --- | --- | --- | --- | --- | --- |
| BS01 | 46 | 1593 | *Thermacetogenium phaeum* (NR_074723.1) | KF990054 | *Firmicutes* | 94% | Syntrophic acetate oxidation | DSM 12270 |
| BS08 | 10 | 1498 | *Thermacetogenium phaeum* (NR_074723.1) | KF990058 | *Firmicutes* | 94% | Syntrophic acetate oxidation | DSM 12271 |
| BS57 | 2 | 1596 | *Thermacetogenium phaeum* (NR_074723.1) | KF990085 | *Firmicutes* | 93% | Syntrophic acetate oxidation | DSM 12272 |
| BS11 | 2 | 1500 | *Thermacetogenium phaeum* (NR_074723.1) | KJ003861 | *Firmicutes* | 93% | Syntrophic acetate oxidation | DSM 12273 |
| BS24 | 2 | 1504 | *[Syntrophaceticus schinkii](http://blast.ncbi.nlm.nih.gov/Blast.cgi#alnHdr_166407389)* (EU386162.1) | KF990067 | *Firmicutes* | 94% | Syntrophic acetate oxidation | Mesophilic anaerobic filter |
| BS72 | 1 | 1496 | *[Syntrophaceticus schinkii](http://blast.ncbi.nlm.nih.gov/Blast.cgi#alnHdr_166407389)* (EU386162.1) | KF990098 | *Firmicutes* | 94% | Syntrophic acetate oxidation | Mesophilic anaerobic filter |
| BS25 | 1 | 1504 | *[Syntrophaceticus schinkii](http://blast.ncbi.nlm.nih.gov/Blast.cgi#alnHdr_166407389)* (EU386162.1) | KF990068 | *Firmicutes* | 94% | Syntrophic acetate oxidation | Mesophilic anaerobic filter |
| BS89 | 1 | 1497 | *[Syntrophaceticus schinkii](http://blast.ncbi.nlm.nih.gov/Blast.cgi#alnHdr_166407389)* (EU386162.1) | KF990112 | *Firmicutes* | 94% | Syntrophic acetate oxidation | Mesophilic anaerobic filter |
| BS75 | 1 | 1504 | *[Syntrophaceticus schinkii](http://blast.ncbi.nlm.nih.gov/Blast.cgi#alnHdr_166407389)* (EU386162.1) | KF990099 | *Firmicutes* | 93% | Syntrophic acetate oxidation | Mesophilic anaerobic filter |
| BS38 | 1 | 1498 | *[Syntrophaceticus schinkii](http://blast.ncbi.nlm.nih.gov/Blast.cgi#alnHdr_166407389)* (EU386162.1) | KF990075 | *Firmicutes* | 94% | Syntrophic acetate oxidation | Mesophilic anaerobic filter |
| BS82 | 1 | 1505 | *[Syntrophaceticus schinkii](http://blast.ncbi.nlm.nih.gov/Blast.cgi#alnHdr_166407389)* (EU386162.1) | KF990105 | *Firmicutes* | 94% | Syntrophic acetate oxidation | Mesophilic anaerobic filter |
| BS55 | 1 | 1505 | *[Syntrophaceticus schinkii](http://blast.ncbi.nlm.nih.gov/Blast.cgi#alnHdr_166407389)* (EU386162.1) | KF990084 | *Firmicutes* | 93% | Syntrophic acetate oxidation | Mesophilic anaerobic filter |
| BS10 | 3 | 1502 | Uncultured *Thermacetogenium* sp. (HQ183800.1) | KF990060 | *Firmicutes* | 93% | Syntrophic acetate oxidation | Leachate sediment in landfill |
| BS21 | 2 | 1502 | Uncultured *Thermacetogenium* sp. (HQ183800.1) | KF990065 | *Firmicutes* | 94% | Syntrophic acetate oxidation | Leachate sediment in landfill |
| BS05 | 1 | 1496 | Uncultured *Thermacetogenium* sp. (JF808029.1) | KF990057 | *Firmicutes* | 90% | Syntrophic acetate oxidation | Produced fluid from Yabase oilfield |
| BS31 | 1 | 1505 | Uncultured *Thermacetogenium* sp. (HQ183800.1) | KF990073 | *Firmicutes* | 93% | Syntrophic acetate oxidation | Leachate sediment in landfill |
| BS35 | 1 | 1499 | Uncultured *Thermacetogenium* sp. (JF808029.1) | KJ003869 | *Firmicutes* | 91% | Syntrophic acetate oxidation | Produced fluid from Yabase oilfield |
| BS28 | 4 | 1485 | *Lutispora thermophila* (NR_041236.1) | KF990071 | *Firmicutes* | 97% | Protein-fermenting | Strain EBR46 |
| BS84 | 1 | 1486 | *Clostridium aldrichii* (X71846.1) | KF990107 | *Firmicutes* | 93% | Cellulolytic | DSM 6159 |
| BS66 | 1 | 1487 | [Uncultured bacterium](http://blast.ncbi.nlm.nih.gov/Blast.cgi#alnHdr_334303184) (JF808030.1) | KF990092 | *Firmicutes* | 99% | — | Produced fluid from Yabase oilfield |
| BS22 | 4 | 1296 | *Anaerobaculum mobile* (CP003198.1) | KJ003866 | *Synergistetes* | 97% | Peptide-fermenting | DSM 13181 |
| BS40 | 1 | 1257 | *Bacteroidetes* bacterium (AY548787.1) | KF990076 | *Bacteroidetes* | 98% | — | Fluidized-bed reactors: acidic wastewater |
| BS70 | 1 | 1503 | Uncultured *Bacteroidetes* bacterium (AB669265.1) | KF990096 | *Bacteroidetes* | 99% | — | Anaerobic digester sludge |
| BS64 | 1 | 1501 | Uncultured bacterium (AB669265.1) | KF990091 | *Bacteroidetes* | 99% | — | Anaerobic digester sludge |
| BS79 | 1 | 1488 | Uncultured *Cytophagales* bacterium (FJ516908.1) | KF990102 | *Bacteroidetes* | 97% | — | The semiarid 'Tablas de Daimiel National Park' wetland |
| BS96 | 1 | 1488 | Uncultured bacterium (AB192126.1) | KJ003880 | *Chlorobi* | 96% | — | Gut homogenate of termites |
| BS58 | 1 | 1490 | Uncultured bacterium (AB192126.1) | KF990086 | *Chlorobi* | 96% | — | Gut homogenate of termites |
| BS94 | 1 | 1494 | Uncultured bacterium (FJ462092.1) | KJ003878 | unclassified | 99% | — | Anaerobic reactor: effluent from the chemical industry |
| BS87 | 1 | 1489 | Uncultured bacterium (EF205585.1) | KF990110 | unclassified | 99% | — | Geothermal spring mat |

OTU, operational taxonomic unit.
